# A Lousy Story

**Published:** 1991-03

**Authors:** J. G. Dumoulin


### From Our Correspondents

### A LOUSY STORY
In World War Two, all Doctors joining the R.A.M.C. attended a course at the Army School of Hygiene. At one lecture we learnt of the newest treatment for body lice. Anti-Louse Powder 63 had secret ingredients which could not be divulged to us less we pass the information to the Germans.
Some time later, I was to put the efficiency of AL63 to the test.

I am now aware that it was about this time that Dr. Kenneth Mellanby was achieving fame for his experiments proving that pediculosis pubis was a venereal disease. Some explanation is therefore required.

At the time I was a medical officer living in the Albanian mountains with the Partisans. There were times when perhaps twenty or more of us would sleep crammed together in a small room. Thus, I got the lice. I bombarded the afflicted area with AL63. To my surprise, the lice thrived on the stuff.

Eventually, I succeeded in getting rid of them by manual removal combined with the liberal use of soap and water.

What some might consider to be blackmail was a ploy used by His Majesty's Armed Forces in those days. If I volunteered to join the Far Eastern section of S.O.E., I could have four weeks home leave. I contacted my fiancée and suggested we get married, to which happily she agreed.

Imagine my distress when a fortnight before the great day I found two little parasites. The medical advice given was that the affected area be shaved completely. But how could I appear on my honeymoon looking like some sex freak! Fortunately, the problem was solved using less drastic treatment, and I was able to report to the church fit and free from infection.

The Germans also had their secret anti-louse weapon. It was invented by no less a person than Hitler's personal physician, Dr. Theodor Morell, described by the Führer as a genius and by Herman Göering as "Herr Reich Injection Master". He was smarter than his English counterpart: he patented his formula which was known as the Morell Russian Lice Powder; and he got Hitler to see that its use was made compulsory for the armed forces. He became a millionaire.

Needless to say, this remedy was also useless.

After a lapse of 50 years, maybe the contents of those powders need be kept secret no longer. Can anyone inform me, please?

J.G. Dumoulin

